# High Throughput Genetic Analysis of Congenital Myasthenic Syndromes Using Resequencing Microarrays

**DOI:** 10.1371/journal.pone.0000918

**Published:** 2007-09-19

**Authors:** Lisa Denning, Jennifer A. Anderson, Ryan Davis, Jeffrey P. Gregg, Jennifer Kuzdenyi, Ricardo A. Maselli

**Affiliations:** 1 Department of Neurology, University of California at Davis, Davis, California, United States of America; 2 Department of Pathology, Medical Investigation of Neurodevelopmental Disorders Institute, University of California at Davis, Davis, California, United States of America; North Carolina State University, United States of America

## Abstract

**Background:**

The use of resequencing microarrays for screening multiple, candidate disease loci is a promising alternative to conventional capillary sequencing. We describe the performance of a custom resequencing microarray for mutational analysis of Congenital Myasthenic Syndromes (CMSs), a group of disorders in which the normal process of neuromuscular transmission is impaired.

**Methodology/Principal Findings:**

Our microarray was designed to assay the exons and flanking intronic regions of 8 genes linked to CMSs. A total of 31 microarrays were hybridized with genomic DNA from either individuals with known CMS mutations or from healthy controls. We estimated an overall microarray call rate of 93.61%, and we found the percentage agreement between the microarray and capillary sequencing techniques to be 99.95%. In addition, our microarray exhibited 100% specificity and 99.99% reproducibility. Finally, the microarray detected 22 out of the 23 known missense mutations, but it failed to detect all 7 known insertion and deletion (indels) mutations, indicating an overall sensitivity of 73.33% and a sensitivity with respect to missense mutations of 95.65%.

**Conclusions/Significance:**

Overall, our microarray prototype exhibited strong performance and proved highly efficient for screening genes associated with CMSs. Until indels can be efficiently assayed with this technology, however, we recommend using resequencing microarrays for screening CMS mutations after common indels have been first assayed by capillary sequencing.

## Introduction

Congenital myasthenic syndromes (CMSs) comprise a distinctive group of disorders in which the normal process of neuromuscular transmission is impaired by one or more pathogenic mechanisms. To date, nine genes have been demonstrated to harbor causative, mostly recessive, mutations for CMSs (Table 1; [1]–[8]). In the majority of these cases, patients present as compound heterozygotes, usually combining a missense mutation in one allele with a missense, nonsense, or frameshift mutation in the other allele of the same gene [4]. Other less frequent defects involve splice junctions [9], promoter regions [10], chromosomal micro-deletions [11], and intronic areas outside the splice junction consensuses [12]. In addition, with few exceptions, mutations responsible for CMSs are private, so that considerable effort is required to detect the mutation or mutations present in each individual. Furthermore, only a few phenotypic clues can point to mutations in one or a limited number of genes [13]. In the absence of these clues, determining the exact genetic causes of CMS in each patient requires that all genes linked to CMSs be amplified and sequenced, a labor and time-intensive undertaking. Thus, there is a real need for a high-throughput technique to efficiently screen the DNA sequences of genes associated with CMSs.

**Table 1:** Genes associated with congenital myasthenic syndromes.

Gene	Symbol	Protein location	Genomic location	Genomic size (bp)	Assayed region size (bp)
*Choline acetyltransferase*	*CHAT*	presynaptic	10q11.2	56,009	3458
*Collagen-like tail subunit of asymmetric acetylcholinesterase*	*COLQ*	synaptic	3p16.2	71,618	3566
*Acetylcholine receptor, alpha subunit*	*CHRNA1*	postsynaptic	2q24-q32	16,861	2019
*Acetylcholine receptor, beta subunit*	*CHRNB1*	postsynaptic	17p13.1	12,526	1977
*Acetylcholine receptor, delta subunit*	*CHRND*	postsynaptic	2q33-q34	9,283	2210
*Acetylcholine receptor, epsilon subunit*	*CHRNE*	postsynaptic	17p13-p12	5,308	5598
*Receptor-associated protein of the synapse*	*RAPSN*	postsynaptic	11p11.2-p11.1	11,413	2227
*Muscle skeletal receptor tyrosine kinase*	*MUSK*	postsynaptic	9q31.3-q32	132,139	3252
*Downstream-of-kinase 7*	*DOK7*	postsynaptic	4p16.2	31,170	Not included

Sequence analysis based on custom resequencing microarrays has recently emerged as a powerful strategy for screening mutations in multiple genes linked to a common phenotype [14]–[16]. This report describes our design and evaluation of a resequencing microarray for mutational analysis of CMSs. We find that with respect to the detection of missense mutations, our microarray performs well. Moreover, it exhibits high specificity and reproducibility. However, this technology is not able to efficiently assay indels. We therefore suggest that resequencing microarrays be employed for mutational analysis after common indels have been screened by capillary sequencing.

## Methods

### Resequencing Microarray Design

Our microarray was designed to sequence all exons and 8 base pairs (bp) of flanking intronic regions from the splice junctions of *CHRNA1*, *CHRNB1*, *CHRND*, *RAPSN*, *COLQ*, *CHAT*, and *MUSK* (Table 1). Additionally, 250 bp of the *RAPSN* and *CHRNE* promoter regions as well as the entire genomic sequence of *CHRNE* were tiled on the microarray. These latter sequences were added because promoter mutations and exonic mutations have been reported in *RAPSN* [5], [14], and promoter, exonic, and intronic mutations have been reported in *CHRNE* [5], [10], [12]. The sequence for each gene was obtained from GenBank (see Table S1) and subjected to Repeat Masker (Institute for Systems Biology, Seattle, WA), a program that identifies repetitive elements (e.g. *SINEs*, *LINEs*, and *ALU*s) and internal duplications. In addition, because the association between CMS and *DOK7* mutations was not known at the time of the design, this gene was not included in the microarray (Table 1).

### Subjects

The sensitivity of the microarray was determined using DNA from 21 CMS patients possessing mutations previously characterized by capillary sequencing. In addition, both the specificity and reproducibility of the microarray were determined using DNA from 5 healthy individuals. This study was approved by the Institutional Review Board of the University of California, Davis. All subjects were informed of their rights and the details of the research, and they all signed an ‘informed consent’ form.

### DNA extraction and PCR

DNA was extracted from blood samples using the QIAamp DNA Blood Mini Kit (Qiagen, Valencia, CA). We used a combination of traditional PCR and long distance PCR to reduce the overall number of reactions required. The size of the PCR amplicons ranged from 170 bp to nearly 13 kb. All primers were designed using Primer3 (http://frodo.wi.mit.edu/cgi-bin/primer3/primer3_www.cgi). Primer sequences and reaction conditions are available upon request.

A 7.5 kb plasmid (IQ-EX) included in the manufacturer's assay (GeneChip® Resequencing Assay Kit, Affymetrix, Santa Clara, CA, USA) was amplified according to the manufacturer's instructions and was used as a positive internal control.

### Quantitation, pooling, fragmentation, and labeling of products

The PCR products were purified of residual reagents using a PCR purification kit (Qiagen) according to the manufacturer's instructions. The DNA concentration of each purified product was measured (ng/µl) (NanoDrop Technologies, Wilmington, DE). After calculating the molarity of each sample, equimolar amounts of the products were pooled to achieve even hybridization across the microarray.

The MicroArray Core Facility at the UC Davis Medical Center (Sacramento, CA) M.I.N.D. Institute performed all of the experimental procedures for the arrays. The pooled PCR products were fragmented using Fragmentation Reagent (0.15U Dnase We/ug DNA, GeneChip® Resequencing Assay Kit, Affymetrix) at 37°C for 15 minutes, followed by inactivation at 95°C for 15 minutes. Pooled and fragmented PCR products were end-labeled using a biotin-labeling reagent (GeneChip® DNA Labeling Reagent, 5 mM, Affymetrix) and terminal deoxynucleotidyl transferase (TdT, Affymetrix) at 37°C for 2 hours, followed by inactivation at 95°C for 15 minutes. The amplified plasmid control (IQ-EX) was fragmented and labeled for use in the hybridization cocktail.

### Microarray Hybridization and Analysis

**Figure 1:**
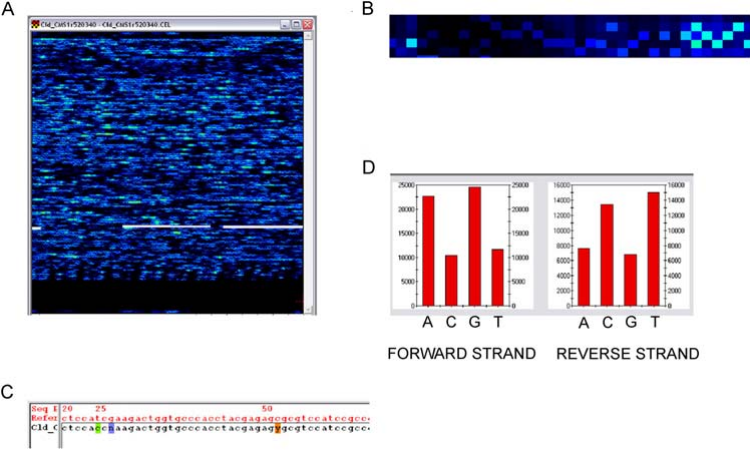
Conversion of microarray probe intensities into sequence by GSEQ v4.0: an illustration of software output. A. A digital color image of the scanned data for the entire microarray. The sense and antisense strands of the DNA fragment are highlighted. B. A close-up view of part of the sense strand highlighted in A. C. The converted sequence displayed in B. D. Probe intensity of the heterozygous site at position 52 shown in C.

Hybridization was performed according to the manufacturer's protocol. The microarrays were placed in a Hybridization Oven 640 (Affymetrix) at 45°C for 16 hours, rotating at 60 RPM. The arrays were then washed and stained on a fluidics station using the manufacturer's wash and stain protocol (DNAARRAY_WS4_450). They were subsequently scanned on a GeneChip® 3000 Scanner (Affymetrix), and the data from each scan were analyzed using GeneChip® Resequencing Analysis Software, Version 4.0 (GSEQ v4.0, Affymetrix). See Figure 1 for an example of the software output.

This software uses an algorithm based on the Adaptive Background genotype Calling Scheme (ABACUS) created by Cutler et al. (2001; [18]). The algorithm allows for 11 possible models: A, C, G, T, AC, AG, AT, CG, CT, GT, and no-call (N). It calculates the likelihood of each model representing the observed data independently for the forward and reverse strands of each position, and it uses these data to calculate an overall likelihood of a particular model fitting the data for that position. There are three results that can arise from the overall likelihood calculation for a particular site: a near perfect fit where the forward and reverse strand fit the same model, an imperfect fit where data from one strand fit the model well but data from the other do not, or a no-call where no model fits the data from either strand. Once an initial call is made, the data must fit reliability rules to ensure the data are reliable; the user has control over certain settings of the reliability rules. A final call is then made for each position. Because some of the reliability rules require that data from each sample be compared to data from other samples at the same position, it is recommended that a minimum of 15 microarrays be analyzed together for optimal algorithm performance.

### Capillary Sequencing

To initially identify the mutations carried by each patient as well as to estimate the percentage agreement between the capillary and microarray sequencing techniques, DNA was amplified as previously described and then sequenced at the UC Davis Division of Biological Sciences DNA Sequencing Facility (Davis, CA) using an ABI 3730 DNA Analyzer (Applied Biosystems, Foster City, CA).

### Analysis

The sensitivity of the microarray methodology with respect to the known pathogenic mutations was defined as the proportion of true positives detected by the microarray, while the specificity was defined as the proportion of true negatives detected. Additionally, the reproducibility was defined as the proportion of identical calls made across the five microarrays assayed with identical DNA. Finally, the percentage agreement between the microarray data and the data produced by capillary sequencing represents the proportion of identical calls between the two methods.

## Results

### Design of the microarray

Screening with RepeatMasker indicated that no repetitive elements or internal duplications were present in the regions resequenced by the microarray. The total number of base pairs resequenced by the microarray was 24,056; 22,214 bp represented genomic sequence covering the eight genes included in the array, and 1,842 bp corresponded to common mutations tiled in duplicate (Tables 1 and 2). An 814 bp internal control, representing the 7.5 kb plasmid control (IQ-EX) provided by the manufacturer was also tiled on the microarray as a means of evaluating individual microarray performance. The microarrays were designed with 25 by 20 micron feature size.

### Sequencing with the CMS1 microarray

**Figure 2:**
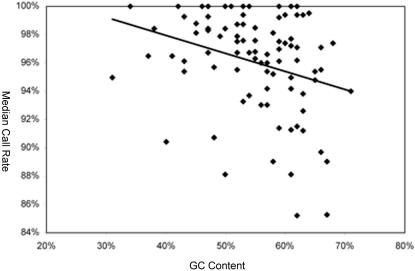
Relationship between GC content and call rate. The median call rate of each fragment across all microarrays is plotted against the GC content of each fragment.

DNA from 26 individuals (21 patients and 5 controls) was sequenced, and for one control, an additional five microarrays were used to determine the reproducibility of the resequencing data. Therefore, a total of 745,736 bases were sequenced across the 31 arrays (21 patient arrays and 10 control arrays; Table 2). The sequence analysis software assigned calls to 698,059 of these bases for an overall call rate of 93.61% (Table 2). Call rates for individual microarrays varied from 92.14%–94.87%. The median GC content of sites assigned a no-call designation (N) for all microarrays was 66%, while the GC content of the entire sequence tiled on the microarray was 57%, a significant difference (*p<*0.01, t-test). A significant negative correlation was also detected between GC content and the median call rate (R^2^ = 0.0873, *p<*0.01; see Figure 2).

**Table 2:** Summary of the microarray data.

Number of base pairs analyzed per chip	24,056
Total number of arrays analyzed	31
Total number of base pairs called by GSEQ	698,059
Overall call rate	93.6%
Overall sensitivity	73.3%
Sensitivity to missense mutations	95.6%
Specificity	100%
Number of chips analyzed for reproducibility	5
Number of base pairs analyzed for reproducibility	113,474
Number of discrepant sites detected among reproducibility chips	1
Overall reproducibility	99.996%

### Findings in patients

According to the capillary sequence data, the 21 patients in this study exhibit a total of 30 mutations, including 23 missense, 4 insertion, and 3 deletion mutations (Table S2). The microarray was able to detect 22 of the missense mutations and none of the insertion or deletion mutations (Table S2). Of the 21 patients, 13 were accurately detected as positive for their respective mutations. All 13 patients were either homozygous or heterozygous for missense mutations. Four additional patients carried one missense mutation and one insertion or deletion mutation, and the missense mutations in these four patients were accurately detected. Three of the 21 patients carried one or two insertion or deletion mutations, none of which was detected by the microarray. Finally, one of the 21 patients was heterozygous for a missense mutation which was not detected by the microarray (Table S2). Overall, the sensitivity of the microarray was estimated to be 73.3%, while the sensitivity of the microarray with respect to missense mutations was estimated to be 95.65% (22/23 detected; Table 2).

### Findings in controls

No pathogenic mutations were detected among the five healthy controls in this study, even though multiple SNPs were detected. Interestingly, a unique nonsynonymous variant was found in a single healthy control. This variant causes an arginine to tryptophan replacement (at site 22 of the amino acid sequence) and was confirmed by capillary sequencing. Although this is a highly conserved residue across mammals, there is no indication this mutation is pathogenic, and the individual was considered to have been correctly identified as a true healthy control. As a result, the specificity of the microarray was 100% (Table 2).

To assess the reproducibility of the microarray approach, five microarrays were hybridized to DNA from the same individual. Calls were assigned to 113,474 of the 120,280 tiled bases, yielding an overall call rate of 94.34% (Table 2). Call rates for each of the five microarrays varied from 93.81%–94.97%, and the average number of calls per microarray was 22,695 out of 24,056. A single discrepancy was found among the five microarrays: two of the microarrays were called ‘G ’, matching the reference sequence, two others were called heterozygotes for ‘A’ and ‘G’, and the last microarray showed a no-call in this position. One discrepancy among an average of 22,695 base calls yielded a reproducibility estimate of 99.996% (Table 2).

### SNPs and comparison between techniques

Heterozygosity estimates have revealed that, on average, SNPs occur at a frequency of 1 in every 1000 bases across the human genome [17]. For this study, a total of 102 SNPs were detected by the microarray, including the 22 missense mutations considered to be pathogenic (Table 3). Of the 80 remaining SNPs, 37 have been documented in the GenBank SNP database (http://www.ncbi.nlm.nih.gov/entrez/query.fcgi?db = Snp; see Table S3). Using the 80, nonpathogenic SNPs, the expected heterozygosity (often referred to as ‘pi’) per site per gene region was estimated (see Table 3). Although there is variation across gene regions, these estimates are close to the genome-wide average of ∼0.001 (Table 3).

**Table 3:** Description of SNPs detected.

Gene	No. microarray SNPs	No. discrepant sites/ No. SNPs checked*	No. new capillary SNPs	Total No. discrepant sites	Expected heterozygosity per site
*CHRNA1*	2	1/2	0	1	0.0002
*CHRNB1*	7	5/7	0	5	0.0015
*CHRND*	11	4/11	0	4	0.0009
*CHRNE*	25	7/21	4	7	0.0009
*RAPSN*	10	1/9	1	1	0.0007
*COLQ*	9	1/9	0	1	0.0007
*CHAT*	10	0/10	0	0	0.0008
*MUSK*	6	1/6	1	1	0.0005
Total	80	20/75	6	20	0.0008

* A total of 75 of the 80 microarray SNPs were cross-checked via capillary sequencing or electronically through the GenBank SNP database.

To determine how well sequence data from the microarray correspond to sequence data from capillary sequencing, 96,686 base calls from across 26 microarrays (21 patients and 5 controls) were compared to capillary sequence data at the same positions. This degree of capillary sequencing allowed us to assess agreement between the two methods for 70 of the 80 SNPs. Of the remaining 10 SNPs, five were verified electronically using the GenBank SNP database (http://www.ncbi.nlm.nih.gov/entrez/query.fcgi?db = Snp). It remains to be determined whether the remaining five SNPs are detected by both sequencing approaches.

A total of 20 discrepant sites were found between the microarray and capillary sequencing data (Table 3). All of these sites involved SNPs detected by the microarray but not by capillary sequencing, and seven of the sites were singletons; that is, they were detected in just a single microarray. Interestingly, six additional SNPs were detected exclusively by the capillary sequencing method, one of which was a singleton; we did not consider these six sites to represent discrepancies, however, as they were called ‘N’s by the microarray (Table 3). None of the discrepant sites called by the microarray is documented in the SNP database, whereas three of the six sites detected by capillary sequencing are corroborated in the database. Overall, 96,635 of the 96,686 assayed bases were identical between the two methods. Therefore, the percentage agreement was 99.947%. However, with respect to sites segregating for SNPs, the percentage agreement between these two methods was only 73.3% (55/75 SNPs; these 55 SNPs are reported in Table S3). Despite this degree of disagreement, estimates of heterozygosity do not change appreciably when using SNP information from the capillary sequencing method (data not shown).

For all of the discrepant sites, the raw trace data from both sequencing techniques were examined. It appears that many of the SNPs called by the microarray software, but not by the capillary technique, were in regions of poor data quality (i.e. several surrounding sites called Ns). In fact, most of the polymorphisms detected in regions of poor data quality were not compelling when examining the raw data, and for many SNP sites, just one of the strands was of good quality (both strands were sequenced for both methods). However, for 8 of the 20 total discrepant sites, convincing and contradictory data were found for both techniques. One approach to addressing this conflict is to employ a third and independent methodology, such as SSCP (single-stranded conformation polymorphism), to re-assay these regions. Alternatively, comparing only data that are validated by a predetermined quality score for both techniques may reveal many fewer, if any, discrepancies [18; see below].

As another approach to assessing the quality of the microarray data, it was determined whether the 80 SNPs exhibited Hardy-Weinberg genotype proportions across the sample. Six of the SNPs were found to deviate significantly from Hardy-Weinberg expectations (*i.e*. χ^2^>3.84, *p<*0.05). To address this, these six loci were re-sequenced in all 26 individuals using capillary sequencing. Overall, departures from Hardy-Weinberg expectations can be explained by the failure of the microarray software to detect all of the heterozygous individuals, either because it called these sites ‘Ns’, or because these sites were called homozygous. In fact, when the genotype proportions provided by the capillary sequence data are used, these SNPs were all found to be in Hardy-Weinberg Equilibrium (data not shown).

## Discussion

Resequencing microarrays provide a rapid and cost effective method for screening mutations in genetically heterogeneous diseases such as CMSs. Indeed, this technique uses many fewer PCR reactions to amplify and sequence long segments of genomic DNA, and it assays multiple genes using a single hybridization reaction. In this study, we were able to analyze more than 24 kilobases of gene regions linked to CMSs from the products of just 35 PCR reactions. In contrast, a capillary sequencing approach requires greater than 100 PCR reactions. Moreover, our resequencing microarray exhibited a high degree of sensitivity with respect to the detection of missense mutations, its average call rate exceeded the 90% rate guaranteed by the manufacturer, it was highly reproducible, and it showed a high level of agreement with the data produced by capillary sequencing.

Although other groups have also reported high performance from custom resequencing microarrays [14]–[16], there are limitations associated with the technology. For example, the call rate of different resequencing microarray designs may vary considerably. In fact, studies have reported anywhere from fewer than 50% of their microarrays achieving a call rate of greater than 90% to nearly 100% of their microarrays achieving a call rate of 97.5% [15], [16]. Importantly, some of this variability can be explained by differences in the user-chosen settings of the CustomSeq™ Algorithm. For example, settings for data filters that capture features with either minimal intensity or intensity approaching the saturation level of the detector can be changed by the user to be more stringent or more relaxed. If the settings are more relaxed, fewer sites will be assigned as no-calls by the filter, but the calls made will be less reliable. If the settings are more stringent, more sites will be assigned as no-calls by the filter and the overall call rate will be reduced, but calls made will be more reliable. Depending on the particular microarray as well as the goals of the study, the optimal set of parameters will vary.

The GC content of a region may also affect the call rate. In fact, our finding that the call rate of the fragments tiled on our microarray decreased as the GC content increased (see Figure 2) corroborates the result of another study, in which 98.4% of the bases assigned as no-calls were either G or C [14]. It is possible that GC-rich probes bind more strongly to the target DNA, thereby increasing the chance of signal saturation and a no-call at a particular position. Interestingly, Cutler et al. (2001; [18]) found that fluorescence intensity declines with the G-richness of a probe, leading to a lower call rate due to the decreased reliability of such probes. However, another study found no correlation between probe content and call rates [19]. Clearly, additional data and analysis are required to understand the relationship, if any, between these two variables.

Despite the high percentage of agreement found in our study, the agreement between the microarray and capillary sequencing techniques with respect to sites segregating for SNPs was only 73.3%. This number is much lower than the overall agreement, in part, because the level of variation in humans tends to be low (i.e. there are many more invariant sites; also see Table 3 for heterozygosity estimates). Consequently, using the microarray technology for high throughput SNP discovery and genotyping has been controversial, and efforts have been made to identify the sites with the highest likelihood of being correct (see [18], and references therein). As mentioned above, this can be accomplished by increasing the stringency settings of the calling algorithm [18]. Accompanying an increase in the reliability of each call, however, is a reduction in the overall call rate. In fact, in their paper introducing the ABACUS algorithm, Cutler et al. (2001; [18]) found that only ∼80% of the sites could be called with these high stringency settings. From our perspective, this may not be an ideal solution, as it is possible that a mutation will be overlooked when screening DNA from a patient with an unknown mutation profile. Such a scenario is of greatest concern for CMS studies when single heterozygous mutations have dominant effects, as in the case of slow-channel syndrome or in rare cases of fast-channel syndrome due to dominant negative mutations [8]. Undoubtedly, the trade-offs between call rate and call quality will continue to be an important issue surrounding the use of resequencing microarrays for mutational analysis.

Another serious limitation of this technology is that it cannot detect insertion and deletion mutations. This is of particular concern for mutations in *CHRNE* and *DOK7*, which together account for a large number of CMS patients, and in which indels are often encountered in the homozygous state. Therefore, we suggest that resequencing microarrays should be used for screening CMS mutations after *CHRNE* and *DOK7* have been screened by capillary sequencing. We hope that the increased capability of the recently available, more powerful 100K and 300K microarray platforms will allow the inclusion of probes complementary to common insertions and deletions at each sequence position to overcome this technological limitation. In fact, such an approach has recently been shown to be feasible [20]. Alternatively, as reviewed by Hacia (1999; [21]), heterozygous indels have been successfully detected when using a loss-of-signal hybridization approach (in contrast to the gain-of-signal approach used in this study). By definition, this approach can only identify the presence of indels; however, capillary sequencing can then be used to determine the actual sequence changes. Clearly, the technology or combination of technologies with the highest power of detection will depend on the particular genes being assayed as well as the goals of the study.

## Supporting Information

Table S1 Genomic Sequences from Genbank.
(0.04 MB DOC)

Table S2 Missense and indel mutations carried by the 21 studied patients.
(0.07 MB DOC)

Table S3 Information on the 55 validated SNPs (also see Table 3).
(0.14 MB DOC)

## References

[1] Engel AG, Sine SM (2005). Current understanding of congenital myasthenic syndromes.. Curr Opin Pharmacol.

[2] Ohno K, Tsujino A, Brengman JM, Harper CM, Bajzer Z (2001). Choline acetyltransferase mutations cause myasthenic syndrome associated with episodic apnea in humans.. Proc Natl Acad Sci U S A.

[3] Ohno K, Brengman J, Tsujino A, Engel AG (1998). Human endplate acetylcholinesterase deficiency caused by mutations in the collagen-like tail subunit (*COLQ*) of the asymmetric enzyme.. Proc Natl Acad Sci U S A.

[4] Engel AG, Ohno K, Sine SM (2003). Congenital myasthenic syndromes: progress over the past decade.. Muscle Nerve.

[5] Ohno K, Engel AG, Shen XM, Selcen D, Brengman J (2002). Rapsyn mutations in humans cause endplate acetylcholine-receptor deficiency and myasthenic syndrome.. Am J Hum Genet.

[6] Chevessier F, Faraut B, Ravel-Chapuis A, Richard P, Gaudon K (2004). *MUSK*, a new target for mutations causing congenital myasthenic syndrome.. Hum Mol Genet.

[7] Beeson D, Higuchi O, Palace J, Cossins J, Spearman H (2006). Dok-7 mutations underlie a neuromuscular junction synaptopathy.. Science.

[8] Webster R, Brydson M, Croxen R, Newsom-Davis J, Vincent A (2004). Mutation in the AChR ion channel gate underlies a fast channel congenital myasthenic syndrome.. Neurology.

[9] Ohno K, Engel AG (2005). Splicing abnormalities in congenital myasthenic syndromes.. Acta Myol.

[10] Nichols P, Croxen R, Vincent A, Rutter R, Hutchinson M (1999). Mutation of the acetylcholine receptor epsilon-subunit promoter in congenital myasthenic syndrome.. Ann Neurol.

[11] Muller JS, Abicht A, Christen HJ, Stucka R, Schara U (2004). A newly identified chromosomal microdeletion of the rapsyn gene causes a congenital myasthenic syndrome.. Neuromuscul Disord.

[12] Muller JS, Stucka R, Neudecker S, Zierz S, Schmidt C (2005). An intronic base alteration of the *CHRNE* gene leading to a congenital myasthenic syndrome.. Neurology.

[13] Beeson D, Hantai D, Lochmuller H, Engel AG (2005). 126th International Workshop: congenital myasthenic syndromes, 24-26 September 2004, Naarden, the Netherlands.. Neuromuscul Disord.

[14] Mandal MN, Heckenlively JR, Burch T, Chen L, Vasireddy V (2005). Sequencing arrays for screening multiple genes associated with early-onset human retinal degenerations on a high-throughput platform.. Invest Ophthalmol Vis Sci.

[15] Tengs T, Lee JC, Guillermo Paez J, Zhao X, Laframboise T (2005). A transforming MET mutation discovered in non-small cell lung cancer using microarray-based resequencing.. Cancer Lett.

[16] Xu N, Podolsky RH, Chudgar P, Chorich LP, Liu C (2005). Screening candidate genes for mutations in patients with hypogonadotropic hypogonadism using custom genome resequencing microarrays.. Am J Obstet Gynecol.

[17] Sachidanandam R, Weissman D, Schmidt SC, Kakol JM, Stein LD (2001). A map of human genome sequence variation containing 1.42 million single nucleotide polymorphisms.. Nature.

[18] Cutler DJ, Zwick ME, Carrasquillo MM, Yohn CT, Tobin KP (2001). High-throughput variation detection and genotyping using microarrays.. Genome Res.

[19] Huentelman MJ, Craig DW, Shieh AD, Corneveaux JJ, Hu-Lince D (2005). SNiPer: improved SNP genotype calling for Affymetrix 10K GeneChip microarray data.. BMC Genomics.

[20] Karaman MW, Groshen S, Lee CC, Pike BL, Hacia JG (2005). Comparisons of substitution, insertion and deletion probes for resequencing and mutational analysis using oligonucleotide microarrays.. Nucl Acids Res.

[21] Hacia JG (1999). Resequencing and mutational analysis using oligonucleotide microarrays.. Nat Genet.

