# Supplementary material for: High Throughput Genetic Analysis of Congenital Myasthenic Syndromes Using Resequencing Microarrays
Source: PLoS One. 2007 Sep 19;2(9):e918. doi: 10.1371/journal.pone.0000918 (PMC1975473; doi:10.1371/journal.pone.0000918)
Supplement: Table S1 — Genomic Sequences from Genbank. (0.04 MB DOC) [file pone.0000918.s001.doc]

Table S1. Genomic sequences from GenBank.

| Gene | Accession Number | Genomic Coordinates |
| --- | --- | --- |
| *CHAT* | NC_000010 | 50487147 – 50543156 |
| *COLQ* | NC_000003 | 15466644 – 15538262 |
| *CHRNA1* | NC_000002 | 175320566-175337427 |
| *CHRNB1* | NC_000017 | 7289130 – 7301656 |
| *CHRND* | NC_000002 | 233099166-233109449 |
| *CHRNE* | NC_000017 | 4741840 – 4747148 |
| *RAPSN* | NC_000011 | 47415891 – 47427306 |
| *MUSK* | NC_000009 | 112470960-112603099 |
