# Supplementary material for: High Throughput Genetic Analysis of Congenital Myasthenic Syndromes Using Resequencing Microarrays
Source: PLoS One. 2007 Sep 19;2(9):e918. doi: 10.1371/journal.pone.0000918 (PMC1975473; doi:10.1371/journal.pone.0000918)
Supplement: Table S2 — Missense and indel mutations carried by the 21 studied patients. (0.07 MB DOC) [file pone.0000918.s002.doc]

**Table S2. Missense and indel mutations carried by the 21 studied patients.**

| *Patient* | *Gene* | *Phenotype* | *Nucleotide change(s)* | *Amino Acid change(s)* | *C** | *M*** |
| --- | --- | --- | --- | --- | --- | --- |
| 1 | *CHRNE* | receptor deficiency | 70insG^†^ | E26X | Yes | No |
| 2 | *CHAT* | deficient acetylcholine re-synthesis | C1642T, C2081G | R548X, S694C | Yes, Yes | Yes, Yes |
| 3 | *COLQ* | ACHE deficiency | T1189A, 1082delC | C397S, L425X | Yes, Yes | Yes, No |
| 4 | *CHAT* | deficient acetylcholine re-synthesis | G580T, G1516T | V194L, V506L | Yes, Yes | Yes, Yes |
| 5 | *CHRNE* | receptor deficiency | IVS1+1G→T, 302insG | NA, T117X | Yes, Yes | Yes, No |
| 6 | *RAPSN* | deficient receptor clustering | G133A, G284A | V45M, E162K | Yes, Yes | Yes, Yes |
| 7 | *CHRNE* | receptor deficiency | 70insG, 1293insG | E26X, D435X | Yes, Yes | No, No |
| 8 | *CHRND* | possible receptor deficiency | T706C^‡^ | C236R | Yes | Yes |
| 9 | *CHRND* | slow channel | C803T^†^ | S268F | Yes | Yes |
| 10 | *CHRNB1* | slow channel | G685T^†^ | V229F | Yes | Yes |
| 11 | *CHRNE* | slow channel | A790C^†^ | T264P | Yes | Yes |
| 12 | *RAPSN* | deficient receptor clustering | C264A, G493A | N88K, V165M | Yes, Yes | Yes, Yes |
| 13 | *CHRNE* | receptor deficiency | 1267delG^‡^ | elongates protein | Yes | No |
| 14 | *CHRNA1* | receptor deficiency | A886C, 459insG | I296L, D347X | Yes, Yes | Yes, No |
| 15 | *CHRNA1* | slow channel | C651G^†^ | N217K | Yes | Yes |
| 16 | *CHRNA1* | slow channel | G457A^†^ | G153S | Yes | No |
| 17 | *CHRNE* | receptor deficiency | G440T, 1198delG | R147I, V407X | Yes, Yes | Yes, No |
| 18 | *RAPSN* | deficient receptor clustering | C264A^‡^ | N88K | Yes | Yes |
| 19 | *CHRNE* | fast channel | C362T, T43C | P121L, Y15H | Yes, Yes | Yes, Yes |
| 20 | *COLQ* | ACHE deficiency | C631T, G1199A | Q211X, C400Y | Yes, Yes | Yes, Yes |
| 21 | *CHRNE* | possible receptor deficiency | T1229G^‡^ | V410G | Yes | Yes |

* ‘C’ reports whether the mutation was detected via capillary sequencing.

** ‘M’ reports whether the mutation was detected via microarray sequencing.

^†^ Heterozygous change

^‡^ Homozygous change
