# Supplementary material for: High Throughput Genetic Analysis of Congenital Myasthenic Syndromes Using Resequencing Microarrays
Source: PLoS One. 2007 Sep 19;2(9):e918. doi: 10.1371/journal.pone.0000918 (PMC1975473; doi:10.1371/journal.pone.0000918)
Supplement: Table S3 — Information on the 55 validated SNPs (also see Table 3). (0.14 MB DOC) [file pone.0000918.s003.doc]

Table S3. Information on the 55 validated SNPs (also see Table 3).

| *Number* | *Gene* | *Gene Coordinate** | *Genome Assembly Coordinate^†^* | **SNP ID Number^‡^** |
| --- | --- | --- | --- | --- |
| 1 | *CHRND* | 385 | 83600722 | ss74804683 |
| 2 | *CHRND* | 392 | 83600729 | ss74804684 |
| 3 | *CHRND* | 2326 | 83602663 | ss74804685 |
| 4 | *CHRND* | 2666 | 83603003 | ss74804686 |
| 5 | *CHRNE* | 331 | 4403442 | ss74804687 |
| 6 | *CHRNE* | 3541 | 4400232 | ss74804688 |
| 7 | *CHRNE* | 3962 | 4399811 | ss74804689 |
| 8 | *CHRNE* | 4053 | 4399720 | ss74804690 |
| 9 | *CHRNE* | 4345 | 4399428 | ss74804691 |
| 10 | *CHRNE* | 4404 | 4399369 | ss74804692 |
| 11 | *RAPSN* | 1310 | 46256662 | ss74804693 |
| 12 | *RAPSN* | 7511 | 46250461 | ss74804694 |
| 13 | *RAPSN* | 7559 | 46250413 | ss74804695 |
| 14 | *RAPSN* | 11262 | 46246710 | ss74804696 |
| 15 | *COLQ* | 67873 | 15435390 | ss74804697 |
| 16 | *COLQ* | 71503 | 15431760 | ss74804698 |
| 17 | *CHAT* | 46034 | 1667638 | ss74804699 |
| 18 | *MUSK* | 26584 | 20778927 | ss74804700 |
| 19 | *CHRNA1* | 14466 | 25824134 | [rs2305416](http://www.ncbi.nlm.nih.gov/SNP/snp_ref.cgi?rs=2305416) |
| 20 | *CHRNB1* | 220 | 6945973 | [rs17856697](http://www.ncbi.nlm.nih.gov/SNP/snp_ref.cgi?rs=17856697) |
| 21 | *CHRNB1* | 2570 | 6948323 | [rs2302765](http://www.ncbi.nlm.nih.gov/SNP/snp_ref.cgi?rs=2302765) |
| 22 | *CHRND* | 5182 | 83605519 | [rs41265127](http://www.ncbi.nlm.nih.gov/SNP/snp_ref.cgi?rs=41265127) |
| 23 | *CHRND* | 5454 | 83605791 | [rs3762528](http://www.ncbi.nlm.nih.gov/SNP/snp_ref.cgi?rs=3762528) |
| 24 | *CHRND* | 9153 | 83609490 | [rs2767](http://www.ncbi.nlm.nih.gov/SNP/snp_ref.cgi?rs=2767) |
| 25 | *CHRNE* | promoter | 4403831 | [rs2302315](http://www.ncbi.nlm.nih.gov/SNP/snp_ref.cgi?rs=2302315) |
| 26 | *CHRNE* | 56 | 4403717 | [rs34563587](http://www.ncbi.nlm.nih.gov/SNP/snp_ref.cgi?rs=34563587) |
| 27 | *CHRNE* | 121 | 4403652 | [rs12602789](http://www.ncbi.nlm.nih.gov/SNP/snp_ref.cgi?rs=12602789) |
| 28 | *CHRNE* | 1468 | 4402305 | [rs33970119](http://www.ncbi.nlm.nih.gov/SNP/snp_ref.cgi?rs=33970119) |
| 29 | *CHRNE* | 4041 | 4399732 | [rs33978919](http://www.ncbi.nlm.nih.gov/SNP/snp_ref.cgi?rs=33978919) |
| 30 | *CHRNE* | 4400 | 4399373 | [rs12940036](http://www.ncbi.nlm.nih.gov/SNP/snp_ref.cgi?rs=12940036) |
| 31 | *CHRNE* | 4483 | 4399290 | [rs12936083](http://www.ncbi.nlm.nih.gov/SNP/snp_ref.cgi?rs=12936083) |
| 32 | *CHRNE* | 4776 | 4398997 | [rs3514](http://www.ncbi.nlm.nih.gov/SNP/snp_ref.cgi?rs=3514) |
| 33 | *CHRNE* | 5082 | 4398691 | [rs1053754](http://www.ncbi.nlm.nih.gov/SNP/snp_ref.cgi?rs=1053754) |
| 34 | *CHRNE* | 5179 | 4398594 | [rs8834](http://www.ncbi.nlm.nih.gov/SNP/snp_ref.cgi?rs=8834) |
| 35 | *CHRNE* | 5210 | 4398563 | [rs7774](http://www.ncbi.nlm.nih.gov/SNP/snp_ref.cgi?rs=7774) |
| 36 | *RAPSN* | 386 | 46257586 | [rs34312154](http://www.ncbi.nlm.nih.gov/SNP/snp_ref.cgi?rs=34312154) |
| 37 | *RAPSN* | 1292 | 46256680 | [rs7111873](http://www.ncbi.nlm.nih.gov/SNP/snp_ref.cgi?rs=7111873) |
| 38 | *RAPSN* | 6447 | 46251525 | [rs34625105](http://www.ncbi.nlm.nih.gov/SNP/snp_ref.cgi?rs=34625105) |
| 39 | *RAPSN* | 10425 | 46247547 | [rs7126210](http://www.ncbi.nlm.nih.gov/SNP/snp_ref.cgi?rs=7126210) |
| 40 | *COLQ* | 42421 | 15460842 | [rs750387](http://www.ncbi.nlm.nih.gov/SNP/snp_ref.cgi?rs=750387) |
| 41 | *COLQ* | 63546 | 15439717 | [rs6782980](http://www.ncbi.nlm.nih.gov/SNP/snp_ref.cgi?rs=6782980) |
| 42 | *COLQ* | 71109 | 15432154 | [rs3846128](http://www.ncbi.nlm.nih.gov/SNP/snp_ref.cgi?rs=3846128) |
| 43 | *CHAT* | 4051 | 1625655 | [rs3729496](http://www.ncbi.nlm.nih.gov/SNP/snp_ref.cgi?rs=3729496) |
| 44 | *CHAT* | 4103 | 1625707 | [rs3810947](http://www.ncbi.nlm.nih.gov/SNP/snp_ref.cgi?rs=3810947) |
| 45 | *CHAT* | 5052 | 1626656 | [rs7903315](http://www.ncbi.nlm.nih.gov/SNP/snp_ref.cgi?rs=7903315) |
| 46 | *CHAT* | 6966 | 1628570 | [rs41306415](http://www.ncbi.nlm.nih.gov/SNP/snp_ref.cgi?rs=41306415) |
| 47 | *CHAT* | 6977 | 1628581 | [rs1880676](http://www.ncbi.nlm.nih.gov/SNP/snp_ref.cgi?rs=1880676) |
| 48 | *CHAT* | 7479 | 1629083 | [rs3810950](http://www.ncbi.nlm.nih.gov/SNP/snp_ref.cgi?rs=3810950) |
| 49 | *CHAT* | 13031 | 1634635 | [rs8178990](http://www.ncbi.nlm.nih.gov/SNP/snp_ref.cgi?rs=8178990) |
| 50 | *CHAT* | 46007 | 1667611 | [rs8178992](http://www.ncbi.nlm.nih.gov/SNP/snp_ref.cgi?rs=8178992) |
| 51 | *CHAT* | 55772 | 1677376 | [rs3793801](http://www.ncbi.nlm.nih.gov/SNP/snp_ref.cgi?rs=3793801) |
| 52 | *MUSK* | 18351 | 20770694 | [rs35142681](http://www.ncbi.nlm.nih.gov/SNP/snp_ref.cgi?rs=35142681) |
| 53 | *MUSK* | 26588 | 20778931 | [rs10980531](http://www.ncbi.nlm.nih.gov/SNP/snp_ref.cgi?rs=10980531) |
| 54 | *MUSK* | 26661 | 20779004 | [rs35176182](http://www.ncbi.nlm.nih.gov/SNP/snp_ref.cgi?rs=35176182) |
| 55 | *MUSK* | 28517 | 20780860 | [rs41279051](http://www.ncbi.nlm.nih.gov/SNP/snp_ref.cgi?rs=41279051) |

*References accession numbers reported in Table S1.

^†^ References the genome reference assembly.

^‡^ Reports the dbSNP ID number. SNPs newly detected in this study are associated only with submitter SNP (ss) accession numbers, whereas already documented SNPs are associated with reference SNP (rs) accession numbers. Reference SNP accession numbers will not be available for the new SNPs until dbSNP Build 128 is released.
